# Is there an increased risk of breast cancer in women who have had a breast cyst aspirated?

**DOI:** 10.1038/bjc.1991.433

**Published:** 1991-11

**Authors:** N. J. Bundred, R. R. West, J. O. Dowd, R. E. Mansel, L. E. Hughes

**Affiliations:** Department of Surgery, University of Wales College of Medicine, Heath Park, Cardiff, UK.

## Abstract

A consecutive series of 644 women who presented with breast nodularity between 1976 and 1982 have been followed up to determine their rate of subsequent breast cancer. Fifteen women have developed breast cancer, 14 of these were among 352 women with an aspirated cyst (relative risk 4.4). Women with multiple cysts had the highest risk and women with breast nodularity had no excess risk. Review of histology specimens from those women who had undergone biopsy showed an excess of florid epithelial hyperplasia in women who subsequently developed breast cancer and women with multiple aspirated cysts were more likely to have florid epithelial hyperplasia. Multiple cysts are clinical markers of histological breast proliferation and women who have had multiple breast cysts aspirated have an increased risk of breast cancer and should be advised to practice regular self examination.


*Br. J. Cancer* (1991), **64**, 953–955 © Macmillan Press Ltd., 1991

### Is there an increased risk of breast cancer in women who have had a breast cyst aspirated?

N.J. Bundred[1], R.R. West[2], J.O. Dowd[3], R.E. Mansel[1] & L.E. Hughes[1]

[1]*Department of Surgery*, [2]*Epidemiology and* [3]*Pathology, University of Wales College of Medicine, Heath Park, Cardiff, Wales, UK.*

**Summary** A consecutive series of 644 women who presented with breast nodularity between 1976 and 1982 have been followed up to determine their rate of subsequent breast cancer.

Fifteen women have developed breast cancer, 14 of these were among 352 women with an aspirated cyst (relative risk 4.4). Women with multiple cysts had the highest risk and women with breast nodularity had no excess risk. Review of histology specimens from those women who had undergone biopsy showed an excess of florid epithelial hyperplasia in women who subsequently developed breast cancer and women with multiple aspirated cysts were more likely to have florid epithelial hyperplasia. Multiple cysts are clinical markers of histological breast proliferation and women who have had multiple breast cysts aspirated have an increased risk of breast cancer and should be advised to practice regular self examination.

Cystic disease of the breast is the most common benign disorder of the breast (Haagensen, 1971), exceeded only by carcinoma in frequency.

Seven per cent of women in the Western World will have a cyst aspirated in their lifetime, but microcystic change on histology is much more common as in 58% of women undergoing breast biopsy (Page *et al.*, 1978) and 23% of women at postmortem (Dupont & Page, 1985) were found to have cystic change. In 1971, Haagensen reported a 4-fold increased risk of breast cancer in women who had undergone cyst aspiration (Haagensen, 1971) but recently two large retrospective studies of patients undergoing breast biopsy for benign disease failed to find any increased relative risk for women who had cystic disease seen only in histological review. (Page *et al.*, 1978; Dupont & Page, 1985). The authors found that only women with histological evidence of proliferative disease (florid epithelial hyperplasia with or without atypia) have an increased risk of breast cancer. (Dupont & Page, 1985).

The differing conclusions reached by these studies led us to examine the long term prognosis of women with benign breast disease. This study seeks to determine if women who have had a breast cyst aspirated have an increased risk of breast cancer, and if so which group of women with cysts have the highest relative risk.

### Subjects and methods

A computerised record of women presenting to the University Department of Surgery Breast Clinic in the University Hospital of Wales has been established since the mid 1970's. Epidemiological information, clinical history of diagnosis are recorded prospectively for all women when they attend. In this study we have retrieved the names of all women with a diagnosis of benign nodularity (formerly fibroadenosis; fibrocystic disease) during the years 1976–1982 (inclusive) from the computer. Six hundred and forty-four women with this diagnosis were seen over this 7 year period. The clinical diagnosis was made by cyst aspiration in 357 women and on clinical mammographic or pathological grounds in the remaining 287 who did not have a cyst aspirated. Both groups have been traced to determine their subsequent breast cancer risk. The method of tracing described by Sims (1973) was used. If hospital notes contained no recent information, the general practitioner and local family practitioner committee were contacted. Finally, the NHS central register was consulted for current general practitioner registration or for details of death. Referrals for breast disease and any other serious illnesses during follow-up were noted.

Correspondence: N.J. Bundred, Department of Surgery, University Hospital of South Manchester, Nell Lane, West Didsbury, Manchester, M20 8LR, UK.
Received 15 January 1991; and in revised form 20 June, 1991.

### Definitions

Aspirable palpable cysts were clinically detected cysts which disappeared on aspiration. Microcysts and macrocysts refer to histopathological entities seen on microscopy.

### Histological review

Histological slides were also available for 91 women (14%) who had undergone breast biopsy either at the initial visit or during follow up. The slides were reviewed for this study by a single pathologist (J.O.D.) without knowledge of the clinical diagnosis, original histological report or subsequent breast cancer development. Histological lesions were classified using the system described by Dupont and Page (1985). Particular attention was paid to the presence or absence of epithelial hyperplasia. When present epithelial hyperplasia was classified as mild, florid or atypical. According to Dupont and Page only florid atypical hyperplasia is associated with increased risk of subsequent breast carcinoma. We made no attempt to quantify the relative amounts of histological complexes within each biopsy specimen.

### Statistical analysis

The expected frequency for breast cancer incidence was calculated in 5 year age groups by multiplying the total woman years in each age group by the age specific incidence rates for breast cancer in England and Wales for the years 1980–86 (O.P.C.S. 1982). Confidence limits for breast cancer risk relative to the general population were calculated using the Poisson distributions. Internal comparison was also made, comparing the relative risks of breast cancer between the cysts and benign nodularity diagnoses by chi-squared tests and by a chi-squared test for trend described by Armitage (Armitage, 1971).

### Results

### Tracing

Of the original 644 women seen with benign nodularity (fibrocystic disease) between the years 1976–1982. Five women (0.8%) were diagnosed as having breast cancer within the first year of follow up and these women have been excluded from further analysis. All women over 30 underwent mammography at initial attendance and two of the five women with cancer within 1 year of the clinical visit had undergone cyst aspiration and were found to have mammographic abnormalities in the contralateral breast which on biopsy proved to be due to invasive ductal carcinoma. The remaining 639 women have been traced via their hospital notes (446, 70%), their original GP (103, 16%) or via the local family practitioner committees to a new GP (44, 7%). Of the remaining women 34 (5%) were traced by writing to the NHS Central Records Department in Southport to determine their current GP. Twelve women (2%) remain untraced.

### Clinical diagnosis grouping

The 352 women who had palpable breast cysts aspirated were significantly older ($P<0.001$) than the 287 women with benign nodularity (and who had never had a cyst aspirated). Multiple cysts were aspirated in 188 women either synchronously or metachronously and 102 women had cysts aspirated bilaterally. In the cyst group 37 (10.5%) women gave a family history of breast cancer in a first degree relative and nine (2.5%) of a family history of cyst formation.

### Incidence of breast cancer

Among the total population of 639 women, 15 (2.3%) developed invasive breast cancer, during a mean 7.2 years of follow-up compared with an expected 5.1 cancers indicating an overall relative risk of 2.9 (95% confidence limits 1.6 and 4.8).

Only one woman in the benign nodularity group developed breast cancer which does not differ significantly from the expected breast cancer rate while 14 women who had cysts aspirated developed breast cancer which was 4.4 times the expected incidence ($P<0.001$ Poisson test) (95% confidence limits 2.4 and 7.3) (see Table I). This increased risk occurred at all ages.

Women with multiple breast cysts were significantly more likely to develop breast cancer than women in either the solitary aspirated cyst group or the benign nodularity group (Chi-squared test for trend = 12.15, $P<0.01$) (Armitage, 1971). Likewise, women who had bilateral breast cysts were more likely to develop breast cancer (Chi-squared test for trend 14.47, $P<0.01$). A family history of breast cancer did not increase the risk of women with aspirated cysts developing breast cancer.

### Patients with breast cancer

Two women out of the 15 patients with breast cancer were diagnosed between 1 and 2 years after first attendance at clinic. The remaining 13 cases were diagnosed between 3 and 12 years (mean 5.1 years) after the first cyst aspiration. One of the two women who developed cancer within 2 years had a past history of having bilateral cyst aspirations some 2 years before at another surgical clinic. She subsequently developed a left sided mammographic abnormality, 13 months after being seen with a right cyst. The other patient had a total of six cysts aspirated from both breasts over a period of 13 months when she was noted to have a residual mass following cyst aspiration. Biopsy proved this to be a 1 cm poorly differentiated invasive carcinoma. In neither case despite review of the hospital notes and mammograms do we feel a breast cancer was missed initially. Six women had undergone bilateral cyst aspiration and developed breast cancer on both sides equally. Seven out of the eight women who had unilateral breast cysts developed breast cancer on the same side as their cyst. Two women presented with impalpable mammographic abnormalities and nine women presented with lumps which were solid on aspiration. Three women had inoperable breast cancer, one had a positive bone scan, one presented with advanced local breast cancer and the third presented to the physicians with postrenal failure secondary to retroperitoneal infiltration by lobular carcinoma. Ten women had node negative carcinoma out of the 12 who underweent mastectomy and axillary clearance. Two women have died from their breast cancer.

**Table I** Incidence of breast cancer on follow-up

| Diagnosis | n | Expected cancers[a] | Observed cancers | Relative risk |
|---|---|---|---|---|
| Benign nodularity | 287 | 1.91 | 1 | 0.52 |
| Aspirated cysts | 352 | 3.18 | 14 | 4.4[b] |

[a]Expected on age specific incidence in England and Wales in years 1980–84 (OPCS 1982–88). [b]Poisson test ($P<0.001$).

### Histology

Histological review of the biopsies from 91 women (14%) revealed 62 (68%) had microcysts, 33 (36%) macrocysts (22 apocrine, 11 non-apocrine) and 64 (70%) apocrine change. Twenty-six (28%) women had mild epithelial hyperplasia, 17 (20%) florid epithelial hyperplasia without atypia and two patients had hyperplasia with atypia, both in the cyst group. (See Table II). Five women who underwent biopsy subsequently developed breast cancer, and all five had apocrine metaplasia and four of five florid hyperplasia on histological review.

Florid epithelial hyperplasia with or without atypia was seen in 19 women and was the only histological change predictive of a subsequent risk of cancer (Chi-squared test for trend = 10.16, $P<0.01$). When the histological findings were correlated with clinical features, women with multiple aspirable cysts were significantly more likely to have florid epithelial hyperplasia in their biopsy specimens (Chi-squared test for trend = 14.63 $P<0.01$, Table II).

### Discussion

The detailed pathological work of Page and Dupont (Page et al., 1978; Dupont & Page, 1985) was widely seen as disproving Haagensen's findings that women who had breast cysts were at an increased risk of breast cancer (Haagensen, 1971). Page and Dupont's study was a histological retrospective review of 10,542 breast biopsies from 3,303 women who were followed up for a mean of 17 years and florid epithelial hyperplasia with or without atypia was the only histological risk factor for subsequent breast cancer development. Their

**Table II** Histological findings in 91 (14%) women who underwent breast biopsy

| | Clinical diagnosis | | | |
|---|---|---|---|---|
| Histological findings | Benign nodularity (n = 19) | Solitary cyst (n = 19) | Multiple cysts (n = 45) | Observed no. of cancers[a] |
| Apocrine change | 17 (63%) | 14 (74%) | 33 (74%) | 5 |
| Epithelial hyperplasia | | | | |
| None | 23 (81%) | 12 (64%) | 14 (30%) | 0 |
| Mild | 4 (15%) | 4 (21%) | 18 (40%) | 1 |
| Florid | 1 (4%) | 3 (16%) | 13 (30%) | 4 |
| Epithelial atypia | 0 | 1 | 1 | 1 |

Florid epitheliosis: Multiple cysts vs nodularity. Test for trend $P<0.01$. [a]Five patients developed breast cancer in the women who underwent biopsy and their biopsies all showed apocrine metaplasia but with varying degrees of co-existing hyperplasia.

incidence of gross cysts on histology was 23% and post-mortem studies suggest an even higher incidence (Davies *et al.*, 1964) yet it is estimated that only 7% of women in the Western world will develop a clinically aspirable breast cyst in their lifetime (Haagensen, 1971). No information is available on the clinical cyst aspiration rate in the Page and Dupont paper. Modern practice is to aspirate breast cysts and histology is rarely available in contrast to the older series (Page *et al.*, 1978; Dupont & Page, 1985; Davies *et al.*, 1964) and thus direct comparison between studies is difficult. Haagensen's original finding of an increased breast cancer risk in women who have had aspiration of a breast cyst has been sustained in this study. Two other clinical studies of breast cancer risk following cyst aspiration have reached similar conclusions (Jones & Bradbeer, 1980; Harrington & Lesnick, 1981). This study is based on patients with aspirated cysts and confirms the previous work (Jones & Bradbeer, 1980; Harrington & Lesnick, 1987 & Haagensen, 1971), but additionally our histological data confirms that florid epithelial hyperplasia with or without atypia is the most important histological risk factor for subsequent breast cancer as did Dupont and Page. Histological macrocysts were common in both groups of women who had been biopsied, but were not a risk factor for subsequent breast cancer unlike clinically palpable cysts. Additionally, women who had undergone multiple cysts aspirations had the highest cancer risk clinically. Thus the best clinical marker for predicting breast cancer risk, multiple cysts, is significantly associated with underlying florid epithelial hyperplasia, the best histological risk marker. This may partly explain the apparent difference in risk of breast cancer between previous clinical and histological studies.

This study showed no increased risk of breast cancer for women who attended the breast clinic with benign nodularity without having a cyst aspirated (generally known as fibrocystic disease) unlike an earlier Cardiff study (Roberts *et al.*, 1984). The excess risk observed in this study is greater than previously reported (Haagensen, 1971; Jones & Bradbeer 1980; Harrington & Lesnick, 1981; Roberts *et al.*, 1984) but other studies have not analysed risk for aspirated cysts separately from biopsy or mammographically proven cysts.

Women with benign nodularity in this study include a group of women with biopsy proven microcystic disease yet they had no increased risk. We must therefore postulate that palpable aspirable cysts are markers of underlying proliferative breast disease and carry a risk in excess of those women with histological microcyst formation, with multiple and bilateral palpable cysts carrying the highest risk. This finding supports our belief that macrocysts should be regarded simply as a normal involutional phenomen (Hughes *et al.*, 1987) whereas cysts that become clinically evident represent real disease.

Although a relative risk of 4.4 times normal appears high it represents only 14 cases in a population of 352 women followed for an average of over 7 years. The majority of the 188 women with multiple cysts were under continued surveillance and had a heightened awareness of the importance of self-examination in which they were well practised (Roberts *et al.*, 1984).

Current surgical practice is to discharge most women who have cysts aspirated from follow-up once they stop forming new cysts. As we have demonstrated an increased risk of breast cancer in this group, a case could be made for continued follow-up but the workload of a surgical outpatients clinic would be significantly increased. A better approach would be to define further sub-groups within the multiple cyst group of women at the highest risk who could then be followed more closely. Possibilities under study include cyst electrolyte analysis (Miller *et al.*, 1983), or serial cyst protein analysis (Haagensen *et al.*, 1979). Another approach would be to suppress cyst formation by drug therapy. Danazol therapy is reported to reduce the number of frequency of new cysts (Dhont *et al.*, 1979) but it is not known whether such therapy has any effect on cancer risk.

Several factors have been shown to be associated with an increased risk of subsequent breast cancer and women who have cysts aspirated should be encouraged to undertake breast self-examination to detect early reappearance of cysts and aid early diagnosis of any cancer that develops.

### References

ARMITAGE, P. (1971). *Statistical Methods in Medical Research*. First Edition, London, Blackwell.

DAVIES, H.H., SIMONS, M. & DAVIES, J.B. (1964). Cystic Disease of the Breast: relationship to carcinoma. *Cancer*, 957.

DUPONT, W.D. & PAGE, D.L. (1985). Risk factor for breast cancer in women with proliferative breast disease. *New England J. Med.*, **i**, 146.

DHONT, M., VAN EYCK, L., DELBEKE, L. & VOORHOOF, L. (1979). Danazol treatment of chronic cystic mastopathy. A chemical and hormonal evaluation. *Post. Med. J.*, **55** (Suppl. 5), 66.

HAAGENSEN, C.D. (1971). *Disease of the Breast*, 2nd Edition: W.B. Saunders and Co.: Philadelphia, 155.

HAAGENSEN, D.E., MAZOUJIAN, G., DILLEY, W.G., PETERSON, C.E., KISTER, S.J. & WELLS, S.A. (1979). Breast gross cystic disease fluid analysis. I. Isolation and radioimmunoassay for a major component protein. *J. Natl Cancer Inst.*, **62**, 239.

HARRINGTON, E. & LESNICK, G. (1981). The association between gross cysts of the breast cancer. *Breast*, **7**, 13.

HUGHES, L.E., MANSEL, R.E. & WEBSTER, D.J.T. (1987). Aberrations of normal development and involution (ANDI). *Lancet*, **ii**, 1316.

JONES, B.M. & BRADBEER, J.W. (1980). The presentation and progress of macroscopic breast cysts. *Br. J. Surg.*, **67**, 669.

MILLER, W.R., DIXON, J.N., SCOTT, W.N. & FORREST, A.P.M. (1983). Classification of human breast cysts according to electrolyte and androgen conjugate composition. *Clin. Oncol.*, **9**, 227.

OFFICES OF POPULATION CENSUSES AND SURVEYS (1982–1988). *Cancer Registration, England and Wales for the years 1980–1984*. H.M.S.O.

PAGE, D.L. VAN DER ZWAGG, R., ROGERS, L.W., WILLIAMS, L.T., WALKER, W.E., HARTMAN, W.H. (1978). Relationship between component parts of fibrocystic disease complex and breast cancer. *JNCI*, **61**, 1055.

ROBERTS, M.M., JONES, ELTON, R.A., FORTT, R.W., WILLIAMS, S., GRAVELLE, I.H. (1984). Risk of breast cancer in women with history of benign disease of the breast. *Br. Med. J.*, **288**, 275.

SIMS, A.C.P. (1973). Importance of a high tracing rate in long term follow up studies. *Lancet*, **ii**, 433.


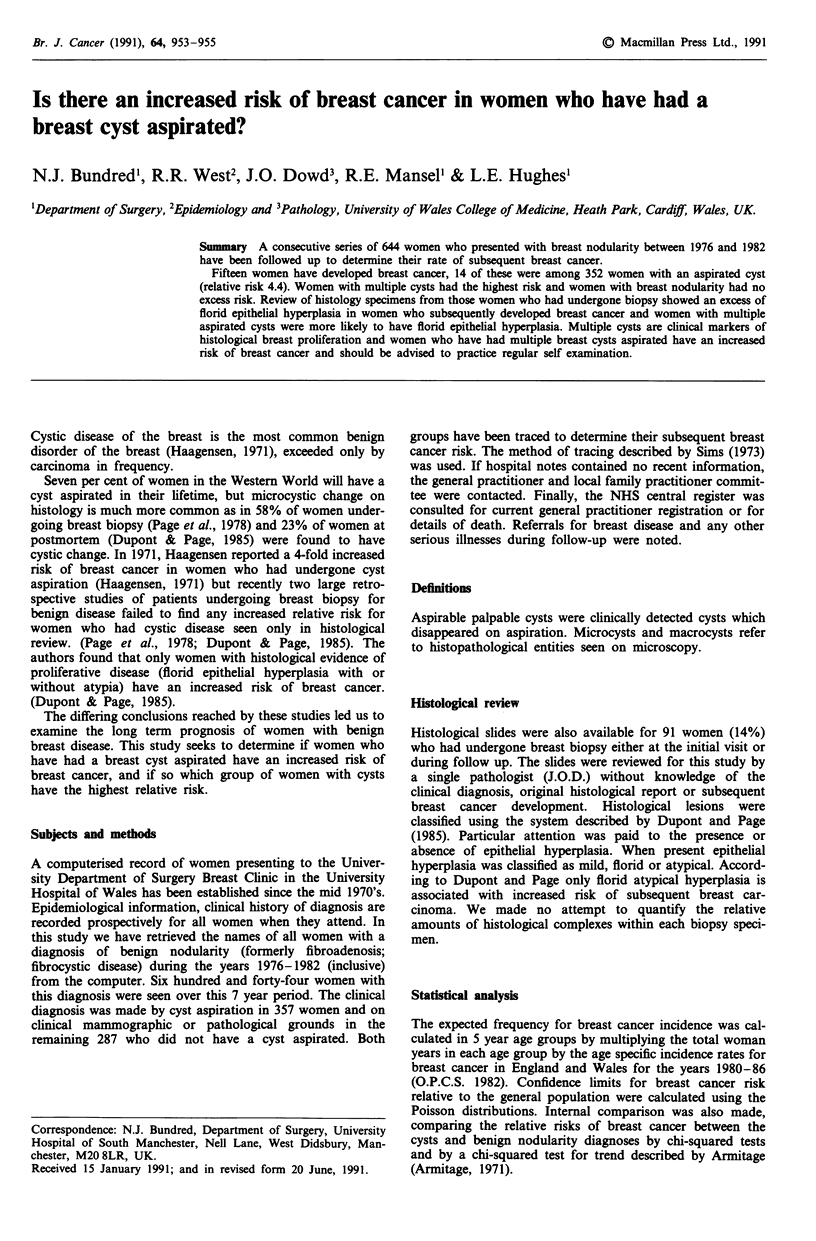

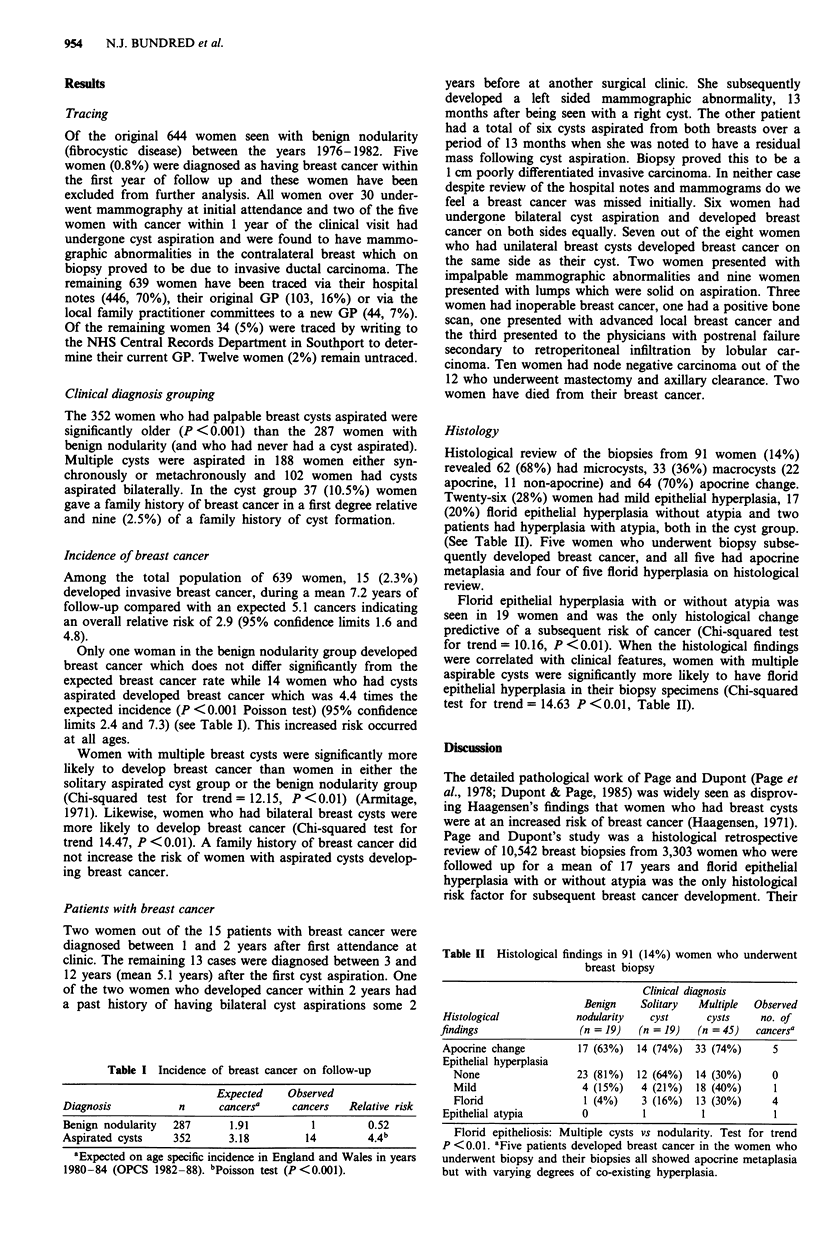

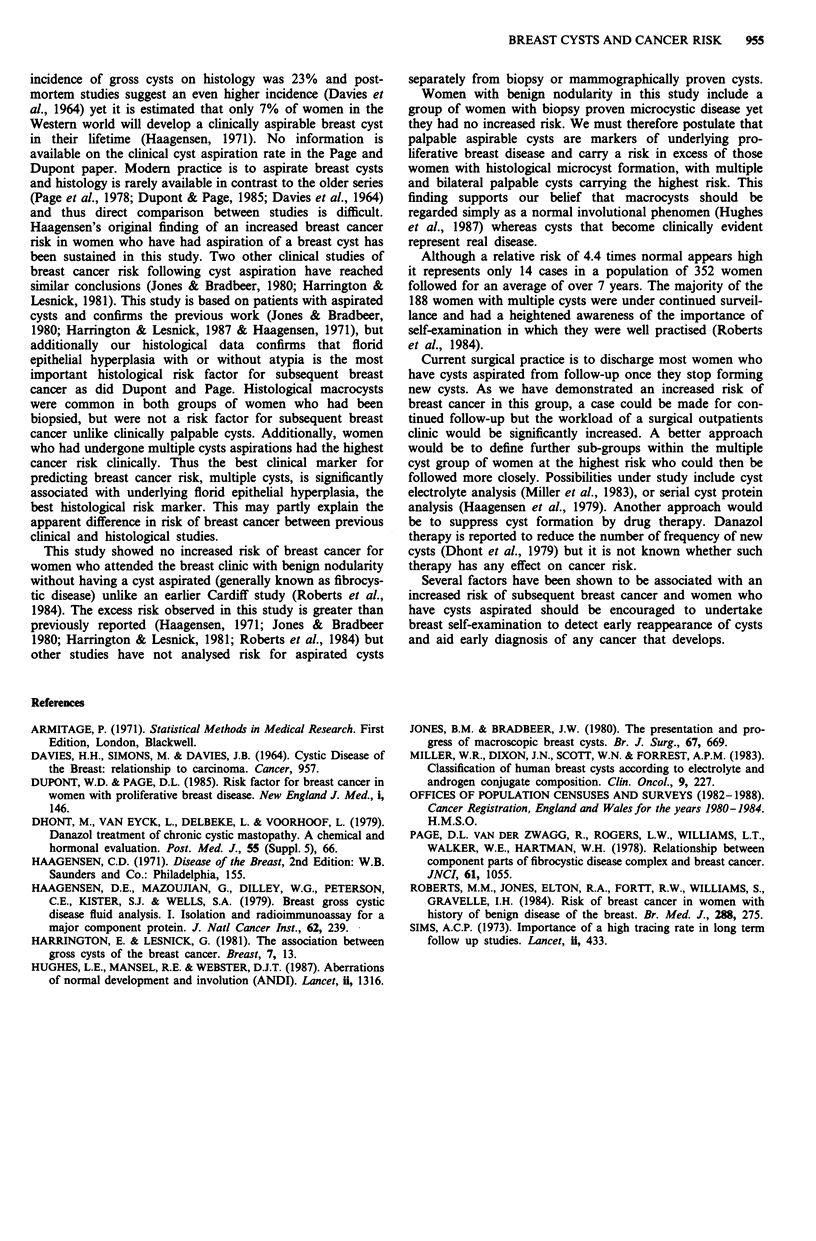


## References

[OCR_00375] DAVIS H. H., SIMONS M., DAVIS J. B. (1964). CYSTIC DISEASE OF THE BREAST: RELATIONSHIP TO CARCINOMA.. Cancer.

[OCR_00384] Dhont M., Delbeke L., van Eyck J., Voorhoof L. (1979). Danazol treatment of chronic cystic mastopathy: a clinical and hormonal evaluation.. Postgrad Med J.

[OCR_00379] Dupont W. D., Page D. L. (1985). Risk factors for breast cancer in women with proliferative breast disease.. N Engl J Med.

[OCR_00393] Haagensen D. E., Mazoujian G., Dilley W. G., Pedersen C. E., Kister S. J., Wells S. A. (1979). Breast gross cystic disease fluid analysis. I. Isolation and radioimmunoassay for a major component protein.. J Natl Cancer Inst.

[OCR_00403] Hughes L. E., Mansel R. E., Webster D. J. (1987). Aberrations of normal development and involution (ANDI): a new perspective on pathogenesis and nomenclature of benign breast disorders.. Lancet.

[OCR_00407] Jones B. M., Bradbeer J. W. (1980). The presentation and progress of macroscopic breast cysts.. Br J Surg.

[OCR_00411] Miller W. R., Dixon J. M., Scott W. N., Forrest A. P. (1983). Classification of human breast cysts according to electrolyte and androgen conjugate composition.. Clin Oncol.

[OCR_00423] Page D. L., Vander Zwaag R., Rogers L. W., Williams L. T., Walker W. E., Hartmann W. H. (1978). Relation between component parts of fibrocystic disease complex and breast cancer.. J Natl Cancer Inst.

[OCR_00429] Roberts M. M., Jones V., Elton R. A., Fortt R. W., Williams S., Gravelle I. H. (1984). Risk of breast cancer in women with history of benign disease of the breast.. Br Med J (Clin Res Ed).

[OCR_00431] Sims A. C. (1973). Importance of a high tracing-rate in long-term medical follow-up studies.. Lancet.

